# Save the world or disappear: the fate of Public Health in an era of formidable challenges

**DOI:** 10.1093/eurpub/ckae090

**Published:** 2024-05-22

**Authors:** Walter Ricciardi

**Affiliations:** Department of Life Sciences and Public Health, Università Cattolica del Sacro Cuore, Roma, Italy

Historians, economists and sociologists are unanimous in saying that there have never been, in the history of humanity, such numerous and complex challenges simultaneously as in the current world.

Pandemics, economic recession, climate change, the collapse of biodiversity, not to mention wars involving more than 2 billion people, including millions of Europeans who thought they had removed this problem forever, are formidable challenges that threaten the very survival of mankind.

The Pope has also long insisted on the fact that ‘this is not an epoch of change, but a change of epoch’ and that pretending nothing has happened will lead to immediate unhappiness.

In the history of public health in past centuries we can identify some well-characterized evolutionary phases:

- Control of infectious diseases (1880–1920)
- Health promotion (1920–60)
- Social engineering (1960–80)
- Health for all (1980–2018)

The 2019 pandemic represented a sharp reversal of trend which, if not stemmed, could lead to a phase of health regression, where health indicators that have constantly improved in recent decades could begin to worsen, some in a way that is difficult to reverse.

As we have seen, the world is becoming more and more complex, people are no longer able to even understand what is happening and often struggle to realize their own ignorance, also because they confine themselves to groups of people with similar ideas, where they exchange information that they validate themselves with a presumption of knowledge that is constantly reinforced and rarely verified.

If you add that most human decisions are based on emotional reactions rather than rational analysis, providing people with more and more precise information is unlikely to improve the situation.

There is no doubt that when, in the past, Hygiene and Public Health actively participated in solving the problems of different historical phases, they played a prominent role, saving millions of lives and improving the health conditions of billions of people.

The main ingredients of this success were: strong scientific and political leadership, great innovative capacity, both from an organizational and technological point of view, extraordinary resilience.

Faced with the new ‘regressive phase’ there is a need to rediscover all these qualities, but it is necessary to add at least one more: courage.

Anti-science activism, particularly in the United States and Europe, is turning into a violent and aggressive movement towards science and scientists.

In his book ‘The deadly rise of anti-science’ the American scientist Peter Hotez describes a phenomenon that now pervades Parliament, the Federal Government, many Governors, the judiciary and the conservative media and has become a dangerous social force that even threatens national security and global stature of the United States.

The anti-science political machine has become a monster of significant proportions and is spreading beyond the pandemic, during which it has already caused enormous damage. Public Health scientists who were previously appreciated and esteemed have become, for large segments of the population, public enemies, paid subjects by the pharmaceutical industry or complicit conspirators of the international elites to gain wealth and power. These feelings are also generated by politicians who tell falsehoods and by the conniving media that spread them, with shocking consequences, such as the over 200 000 Americans who died because they believed the anti-scientific theories of anti-vaxers and the politicians who supported them, killing more citizens than everyone else those victims of firearms, terrorism, nuclear proliferation and cyber attacks and COVID-19 represented a catalytic event that broadened and amplified the rejection of vaccines and science.

Since silence is not neutral, scientists should get involved and participate more assiduously in public debates and should not be afraid to make their voices heard loud and clear when the debate falls within their fields of experience.

As historian Harari underlines, ‘It is certainly vital to make progress in academic research and publish the results in scientific journals that only a small number of experts read, but it is equally essential to communicate the most up-to-date scientific theories to the general public with popular scientific books and even through the competent use of “art and narration”’.

Countering anti-scientific attacks requires new approaches to science communication and public engagement. Particularly in the biomedical sciences we need a new generation of scientists who are committed to defending science in public places and, sometimes, defending colleagues from attacks by politicians, the media and pseudo-intellectuals.

In other words, courageous public health professionals.

The game is epochal: if we succeed, we could literally contribute to saving the world as we have known it in the past millennia. If we fail, we will be condemned, together with our beloved discipline, to irrelevance.

It is a challenge that we must win.

*Conflicts of interest*: None declared.

## References

[1] Harari YN. 21 Lessons for the 21st Century. New York, USA: Penguin Random House, 2018.

[2] Hotez PJ. The deadly rise of anti-science. A Scientist’s Warning. Baltimore, USA: Johns Hopkins University Press, 2023.

